# Recent advances of edible marine algae-derived sulfated polysaccharides in antiviral treatments: challenges vs. opportunities

**DOI:** 10.3389/fnut.2025.1561119

**Published:** 2025-03-26

**Authors:** Xiaoying Dong, Yusong Qiu, Nan Jia, Yinfeng Wu, Qing Nie, Jiahui Wen, Chao Zhao, Yongzhen Zhai

**Affiliations:** ^1^Department of Infectious Disease, Shengjing Hospital of China Medical University, Shenyang, China; ^2^State Key Laboratory of Mariculture Breeding, Key Laboratory of Marine Biotechnology of Fujian Province, Fujian Agriculture and Forestry University, Fuzhou, China; ^3^College of Marine Sciences, Fujian Agriculture and Forestry University, Fuzhou, China; ^4^College of Marine and Biology Engineering, Yancheng Institute of Technology, Yancheng, China

**Keywords:** marine polysaccharides, sulfated polysaccharides, virus, antiviral mechanisms, algae

## Abstract

Marine polysaccharides, particularly those derived from red, brown, and green algae, have shown promising antiviral activity. Among them, sulfated polysaccharides are particularly notable due to their broad-spectrum antiviral properties. These include direct viral destruction, inhibition of virus adsorption, disruption of viral transcription and replication, and the stimulation of the host’s antiviral immunity. With low toxicity, minimal drug resistance, and excellent biocompatibility, these polysaccharides represent promising candidates for the development of antiviral medications. For instance, carrageenan, a polysaccharide from red algae, and fucoidan, a polymer from brown algae, have both been proven to effectively inhibit viral infections. Sulfated polysaccharides from green algae, such as those found in *Ulva* species, also exhibit antiviral properties, including activity against the Japanese encephalitis virus. These polysaccharides function by blocking the attachment of viruses to host cells or interfering with various stages of the viral life cycle. Moreover, marine polysaccharides have been shown to enhance host immune responses, thereby aiding in viral clearance. Although these findings highlight the antiviral potential of marine polysaccharides, most studies have been conducted *in vitro* or in animal models. Further clinical trials are necessary to validate their effectiveness and safety for therapeutic use.

## 1 Introduction

**Figure 1:**
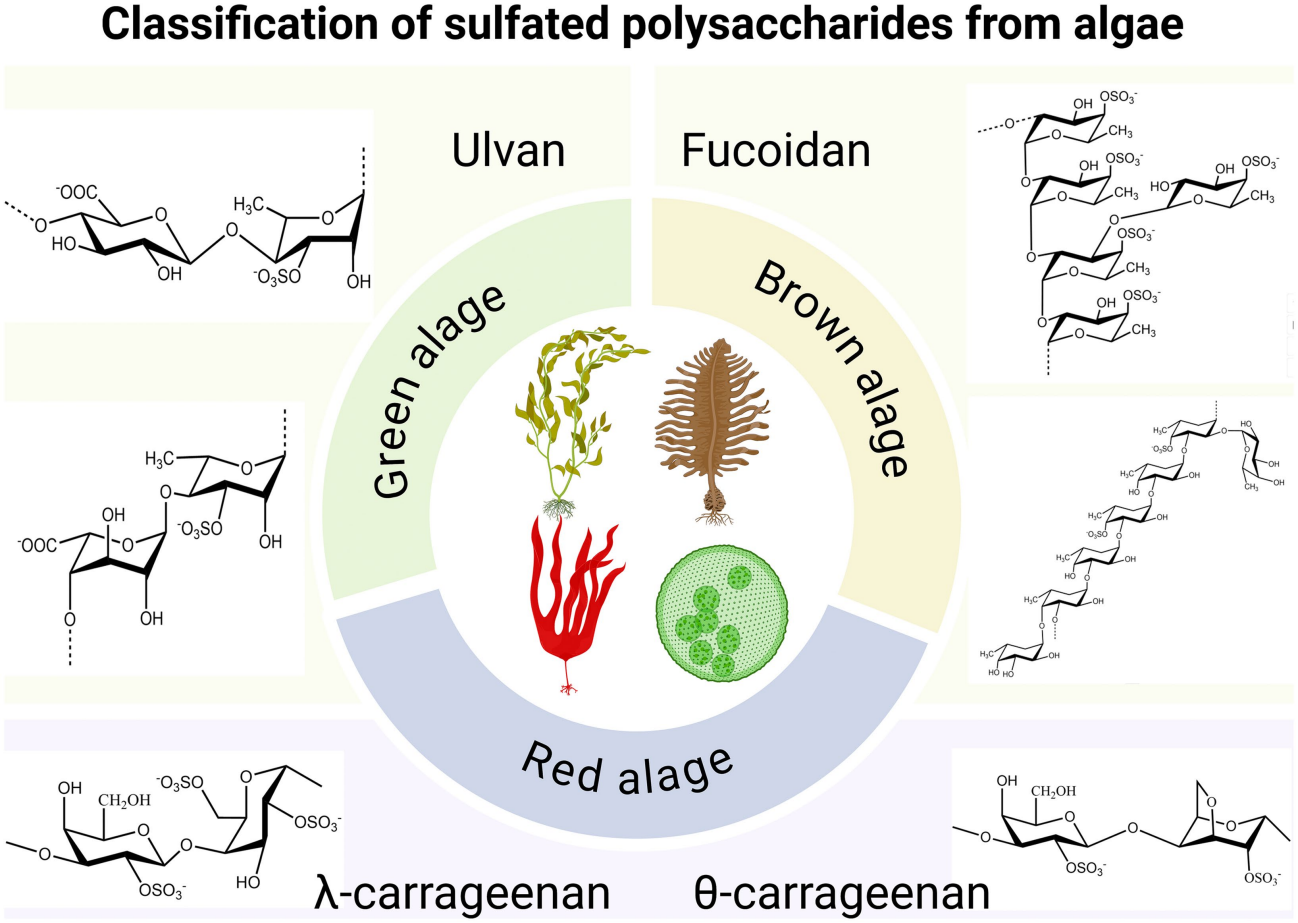
Classification of sulfated polysaccharides from algae. Created with BioRender.com.

Algae are photosynthetic organisms widely distributed in nature. Based on their size and structure, they can be categorized into microalgae and macroalgae. Microalgae usually exist as single cells or in small groups, whereas algae exhibit multicellular structures and mainly include the categories of green, brown and red algae (1). At present, researcher have found antiviral, anti-tumor, anti-inflammatory, antibacterial, immune regulation, hypoglycemic, hypolipidemic and other active substances in algae. Among them, obtaining new antiviral drugs from algae has attracted extensive attention (2–10). In recent years, antiviral substances derived from algae have primarily focused on seaweed polysaccharides, proteins, small molecules, and algae extracts (Table 1). Among these, the sulfated polysaccharides from algae have been the subject of the most extensive research (Figure 1), which showcases the structural classification of sulfated polysaccharides derived from red, green, and brown algae (11). These polysaccharides can inhibit viral invasion and replication by interfering with the binding of viruses to receptors on the surface of host cells, making them a strong candidate for the development of natural antiviral drugs (12).

**Table 1:** Antiviral activity of non-sulfated polysaccharides.

Antiviral active substances	Sources	Virus types	Mechanisms	References
Cyanobacterial lectin *Oscillatoria agardhii* agglutinin	*Oscillatoria agardhii strain*	HIV (Human Immuno-deficienc-y Virus)	/	(118–120)
Cyanovirin-N	*Cyanobacteria Nostoc ellipsosporum*	SIV (Simian Immuno-deficienc-y Virus), HIV	Block gp120 interaction with the CD4 + receptor, the chemokine CCR5, or the CXCR4 coreceptor.	(121–123)
Ethanolic extract	Microalgae *Chlorella*	HSV-1 (Herpes Simplex Virus type 1)	Direct virus killing effect	(124, 125)
Neoagarohexaose (NA6)	Red algae	Noroviru-ses	Induce TLR4-TRIF pathway, produce immune response and reduce norovirus replication.	(126, 127)
Palmitic acid	*Sargassum* (SP4-2)	HIV-1 (Human Immuno-deficienc-y Virus type 1)	Inhibit virus attachment without damaging CD4 receptor on cell surface	(128)
Protein-binding pigment	Blue-green algae	EV71 (Enterovi-rus 71)	Delay the synthesis of viral RNA in infected cells, reduce the process of apoptosis, reduce membrane damage and regulate cell cycle	(129)
Semi-refined extract	*Arthrospirochaeta platensis*	HSV (Herpes Simplex Virus), CTMV (Cytome-galovirus)	/	(130)
SP4-2 (*Sargassum fusiforme* polysaccharide fraction)	*Sargassum fusiforme*	HIV-1	Blocking the entry of virus and efficiently inhibiting the replication of virus after entry	(131–133)
Sulfoquinoline diacylglycerol (SQDG)	Red alga *Pseudomonas Aeruginosa*; microalgae *Spirulina platensis*; Green algae *Porphyra racemose*; Brown algae *Sargassum vulgare*	HSV-1, HSV-2 (Herpes Simplex Virus type 2)	/	(134–137)

**Figure 2:**
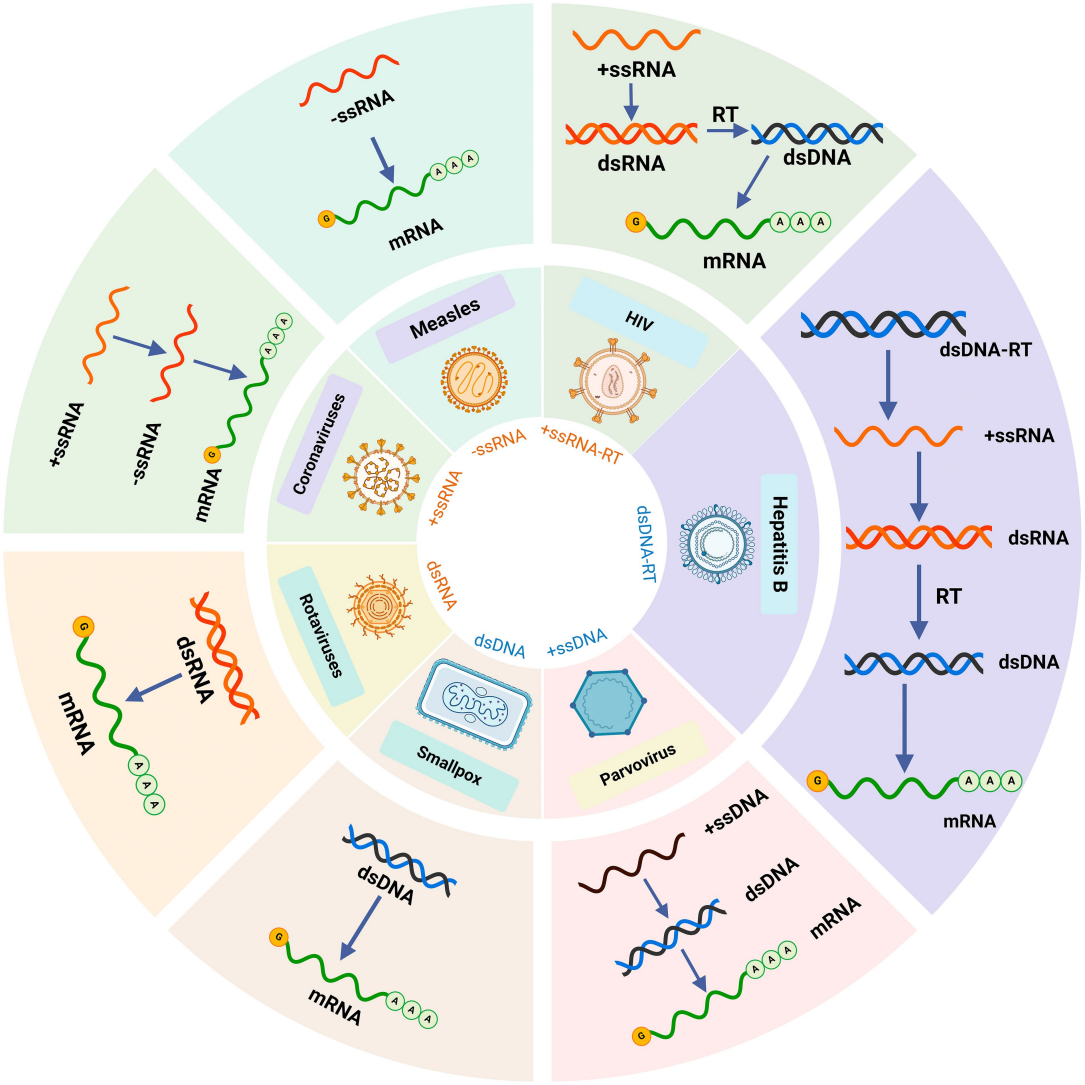
The Baltimore classification of viruses. Created with BioRender.com.

Viruses, which are pathogenic microorganisms that pose significant threats to human health and life, are classified in the Baltimore classification system (Figure 2). About 60% of infectious diseases are caused by viruses (13). They are unique in that they can only reproduce inside other organisms’ cells. Viruses are generally assumed to be inactive since their genetic material is mostly a type of DNA or RNA, which has a protein shell and relatively simple structure (14). The primary method for preventing viral diseases is vaccination. However, vaccines for some viruses are still not available (15). In addition, some of the virus replication processes are within the cellular metabolic pathway and it is therefore not simple to eradicate virus particles (16). The antiviral drugs used are toxic and cannot completely eliminate the virus, and some even cause serious virus resistance (17). Therefore, it is an extremely urgent task to find and develop new natural antiviral substances with different mechanisms and low toxicity. This paper aims to provide a comprehensive review of the antiviral potential of marine sulfated polysaccharides, particularly those derived from green, brown, and red algae. The focus is on the structure–function relationship of these polysaccharides, including the key monosaccharides and sulfation patterns that contribute to their antiviral activity. By examining the molecular mechanisms through which these polysaccharides exert their effects, we seek to enhance the understanding of their therapeutic potential, highlighting their promise as natural antiviral agents with low toxicity and minimal drug resistance, while also emphasizing the need for further clinical studies to validate their efficacy in human applications.

## 2 Methodology

This study uses Google Scholar, PubMed Pro, and Web of Science to search for the literature of algal polysaccharides. Advanced search includes keywords in the keywords, abstract, and title of the articles. The keywords used are “antiviral - algal polysaccharide,” “algal polysaccharide,” “activity - algal polysaccharide,” and “structural composition - algal polysaccharide” with particular focus on antiviral mechanisms of algal polysaccharides. Non-English articles are excluded to ensure consistency in language. Additionally, further exploration of terms such as “algal polysaccharide antiviral mechanism,” “immune response modulation by algal polysaccharides,” and “mechanism of green algal polysaccharides against viral infection” is undertaken to capture a broader range of relevant research on the antiviral properties and immune-modulating effects of algal polysaccharides.

## 3 Antiviral studies on sulfated polysaccharides

Sulfated polysaccharides is a name given to naturally and semi-synthetic acid polysaccharides formed by substituting the hydroxyl group on the monosaccharide in the macromolecular chain with a sulfate group (18). Antiviral activity is thought to be one of the most significant biological activities of sulfated polysaccharides (Table 2), whether they are synthesized by direct extraction or by chemical modification (19). The selection of an appropriate extraction method for sulfated polysaccharides depends on the desired structural integrity and biological activity of the extracted compounds. For instance, carrageenan, derived from red algae such as *Kappaphycus alvarezii* and *Eucheuma denticulatum*, is typically extracted using hot water extraction, alkali treatment, and enzymatic hydrolysis (20). Fucoidan, a sulfated polysaccharide from brown algae, is commonly extracted from *Fucus vesiculosus*, *Laminaria japonica*, and *Sargassum* spp. using acid extraction, enzymatic hydrolysis, and ultrasound-assisted extraction (21). Ulvan, originating from green algae such as *Ulva lactuca* and *Enteromorpha prolifera*, is usually obtained through hot water extraction, acid or alkali treatment, and microwave-assisted extraction. Currently, commonly employed extraction techniques include hot water extraction, microwave-assisted extraction, and ultrasound-assisted extraction (22).

**Table 2:** Antiviral activity of sulfated polysaccharides from various sources.

Sulfated polysaccharides	Sources	Virus types	Mechanisms	References
Ascophyllan	*Ascophyllum nodosum*	HIV, HBV (Hepa-titis B Virus), HCV (Hepatitis C Virus)	Suppress the initial stage of viral infection	(138)
DS-1, DS-2e, DS-3	*Cryptonemia seminervis*	HMPV (Human Metapneumovirus)	Block viral replication by attaching to the viral particle; prevent HMPV from recognizing cell receptors and entering the host cell	(139)
Fucoidans	*Sargassum mcclurei, Turbinara ornata, Sargassum polycystum,*	HIV	Inhibit the initial stages of HIV entry into target cells	(140)
GiWE	*Grateloupia indica*	DENV-2 (Dengue Virus type 2)	Disrupt viral adsorption and internalization	(141)
Heparin	/	SARS-CoV-2 (Severe Acute Respirator-y Syndrome Coronaviru-s 2)	Attach to the receptor-binding domain of the Spike protein and trigger a conformational change	(142)
Iota-carrageenan	*/*	HRV (Human Rhinovirus), H1N1 (Influenza A virus H1N1), hCov (human Coronaviru-s), hMPV (human Metapneumovirus)	Bind to and render virus particles inactive; Inhibit the release of viruses from infected cells	(143, 144)
Fucoidan (KW)	*Kjellmaniella crassifolia*	IAV (Influenza A Virus)	Attach to viral neuraminidase (NA) and suppress its activity, thereby preventing viral release	(69)
P1S, P2S	*Azadirachta indica*	HSV-1	Interfere with the initial stages of viral replication, including the adsorption process	(145)
PAE	*Entermorpha intestinalis*	RSV (Respirator-y Syncytial Virus), IBV(Infecti-ous Bronchitis Virus), HSV-1/−2, Enterovirus 71	/	(146)
p-KG03	*Gyrodinium impudium*	H1N1, H3N2 (Influenza A virus H3N2)	Block viral attachment, cellular entry, and initial viral replication stages	(147)
sABPS	*Achyranthes bidentata*	PRRSV (Porcine Reproducti-ve and Respiratory Syndrome Virus)	Inhibit viral adsorption and replication	(23)
sAPS	*Astragalus membranaceus*	IBDV (Infectious Bursal Disease Virus)	Bind to viruses or cells, obstruct virus adsorption, or inhibit some steps in viral replication post-entry	(148)
sBSRPS	*Bush sophora* root	DHAV-1 (Duck Hepatitis A Virus type 1)	Inhibit the translation of viral proteins and the synthesis of RNA	(149)
SCPPS-1	*Codonopsis pilosula*	HSV-1	Occupy the receptor binding sites, leading to a reduction in viral adsorption; affect the functions of key enzymes involved in viral replication	(150)
ShWE	*Scinaia hatei*	HSV-1/−2	Inhibit viral replication	(65)
sRCP	*Cyathula officinalis*	HSV-2	Hinder the virus adsorption process	(151)

Sulfated polysaccharides were identified to effectively inhibit some viruses, including influenza A virus (IAV), human immunodeficiency virus (HIV), dengue virus (DENV), herpes simplex virus (HSV) and hepatitis B virus (HBV) (23–25). In addition, sulfated polysaccharides are revealed to be of low cytotoxicity and drug resistance, show good biocompatibility with zero side effects, and are reported to activate immune functions (26–28). Hence, it provides a point of departure for designing new food supplements and antiviral medicines.

Extracting sulfated polysaccharides from algae has become an important method for obtaining natural sulfated polysaccharides (29, 30). The content of natural sulfated polysaccharides in algae is extremely rich (12). Common sulfated polysaccharides include fucoidan, which is derived from kelp and brown algae, as well as carrageenan and agarose. Additionally, sulfated mannan, sulfated polysaccharides from green algae (particularly *Ulva pertusa*), and various sulfated polysaccharides extracted from *Spirulina* are also notable. These naturally occurring polysaccharides exhibit varying degrees of antiviral activity, as summarized in Table 3.

**Table 3:** Antiviral activity of algal sulfated polysaccharides.

Sulfated polysaccharides	Sources	Virus types	Mechanisms	References
Alginate polysaccharides	Brown algae	HIV	Inhibiting viral reverse transcriptase and interfering with viral adsorption	(112)
Carrageenan oligosaccharide KCO	*Chondrus, Gigartina, Hypnea, and Eucheuma,* Rhodophyta	Influenza A virus (IAV)	Inhibition of IAV replication before virus release	(49)
Ca-SP	*Spirulina platensis*	HIV-1, HSV-1	Inhibition of virus entry into host cells	(152)
Focans	*Dictyota mertensii, Lobophora variegata, Spatoglossum schroederi* and *Fucus vesiculosus*	HIV	Inhibit the activity of HIV reverse transcriptase	(113)
Fucoidan	Brown algae	HIV, HSV and human cytomegalovirus	Suppress the binding of virus particles to host cells	(153)
Fucoidans SHAP-1 and SHAP-2	Brown alga *Sargassum*	HSV-2	Inhibit the adsorption of HSV-2 virions to host cells	(57)
*Laminaran*	Brown seaweeds	HIV	Inhibition of HIV adsorption on lymphocytes and HIV reverse transcriptase activity	(154)
Lota-carrageenan	*Undaria pinnatifida* and *Splachnidium Rugosum*, Red algae	Human metapneumonic virus (hMPV)	Prevent the release of viruses from the cell membrane and inhibit viral infection	(143)
PMGS Phlorotannin-modified Gracilaria sp. polysaccharides	Brown algae	HPV (Human Papillomavirus), HIV-1	Suppress the adsorption and entry of HIV-1	(146)
Ulvan	Green algae	NDV (Newcastle Disease Virus), HSV, VSV (Vesicular Stomatitis Virus)	Inhibition of VSV infection and replication	(155)
ι-carrageenan	*Chondrus, Gigartina, Rhodophyta, Hypnea, Eucheuma*	HIV, DENV (Dengue Virus)	Inhibiting virus replication	(48, 156)
*λ*-carrageenans	*Sphaerococcus coronopifolius, Rhodophytha, Boergeseniella thuyoides, Rhodophyta*	HIV, HSV	Inhibit HSV-1 and HIV-1 replication	(157)

### 3.1 Sulfated polysaccharides extracted from red algae

The red algae that contain sulfated polysaccharides mainly include *Chondrus*, *Euchema*, *Furcellaria*, *Gigartina*, *Hypnea*, and *Iridae*. The major cell wall component of red algae is a class of sulfated linear polysaccharides, also called carrageenan, which accounts for over 30%, and even up to 75%, of the dry weight of the algae (31–35). It has a repetitive disaccharide structure in carrageenan, with alternating 4-linked 3,6-anhydro-*α*-D-galactopyranose or 4-linked α-D-galactopyranose and 3-linked *β*-D-galactopyranose, and are substituted with sulfates at various positions (36, 37). The lambda-carrageenan is composed of D-galactose units, which have nearly three equatorial sulfates. The iota-and kappa-carrageenan are made up of an equal amount of alternating galactose and 3,6-anhydrogalactose kappa-with a sulfate group while iota-contains two disaccharides in the axial position (38).

Based on their structural characteristics, such as sulfation patterns and the presence of 3,6-anhydro bridges in *α*-linked galactose residues, carrageenans are classified into various types designated by Greek letters: kappa (*κ*)-, iota (*ι*)-, lambda (*λ*)-, nu (*ν*)-, mu (*μ*)-, and theta (*θ*)-carrageenan (39, 40). In addition to alternating sugar units and substituted sulfates, carrageenans also contain other carbohydrate residues like glucose, uronic xylose, and acid (41, 42). Various unique physical and chemical properties are related to the structure and composition of different types of carrageenans (43). According to their unique and different properties, carrageenans are widely used in food, cosmetics and pharmaceutical industries (42). Different types of carrageenans also exhibit many biological characteristics, such as antiviral, antithrombotic, anticancer and immunomodulatory activities (44). The *κ*-, *ι*-and *λ*-carrageenans are three kinds of carrageenans with deeper research and greater economic development value, which have better inhibition effects on different viruses (45). Different types of carrageenans or modified carrageenans isolated from various algae have not been detected to be toxic. In many cases, carrageenan treatment does not have harmful effects on metabolic activity or cell morphological, and no irritation or toxicity has been observed in the *in vivo* experiments, all of which prove that carrageenan is a non-toxic additive and has been approved by the European Union for use as a food additive (46, 47).

Research has shown that, *ι*-and *λ*-carrageenan exhibit effective inhibitory effects on dengue virus type 2 and 3 in Vero and HepG2 cells, with effective concentration of 0.14–4.1 μg/mL (48). This inhibitory effect on the virus is achieved by reducing the number of plaques to suppress virus production, and does not depend on the multiplicity of the multiplicity of infection (MOI) in the range of 0.001 to 1. λ- Carrageenan has a dual interference and inhibitory effect on virus adsorption and nucleocapsid internalization into cytoplasm. A study has shown that the 2 kDa *κ*-carrageenan oligosaccharide derived from carrageenan polysaccharides effectively inhibits the replication of H1N1 influenza virus in Madin-Darby Canine Kidney (MDCK) cell, with a selectivity index greater than 25.0 (49). In addition, 2 kDa *κ*-carrageenan oligosaccharide better inhibited the replication of influenza A virus than the higher molecular weight κ-carrageenan oligosaccharide, and showed a dose dependent inhibitory effect on IAV proliferation. The anti-virus effect of κ-carrageenan oligosaccharide may directly inactivate virus particles after pretreatment of MDCK cells. Unlike carrageenan polysaccharide, κ-carrageenan oligosaccharide can also prevent the expression of IAV mRNA and protein after internalization into cells, but it does not interfere with virus adsorption.

### 3.2 Fucose sulfated polysaccharide extracted from brown algae

Alginate, a prominent acidic polysaccharide found extensively in the cell walls of brown algae, consists of poly-D-glucuronic acid, the central skeleton of poly-D-mannuronic acid, the alternating residues of D-couric acid and D-mannuronic acid (50). Alginate is widely utilized in biomedical science and engineering for its antiviral properties (49). Fucoidan is a sulfated polysaccharide characterized by fucose skeleton (51), and is the most widely studied and applied fucose in brown algae (52). It was first separated by Kylin in 1913 (53). Early studies on its structure showed that the main chain of fucoidans from brown algae in the order Fucales (family Fucaceae) is composed of alternating 1 → 3- and 1 → 4-linked *α*-L-fucosyl residues (54–56). Studies have shown that the fucoidan SHAP-1 and SHAP-2 separated and purified from *Sargassum* have anti HSV activity. Both fucoidan SHAP-1 and SHAP-2 are composed of galactose and fucose. The skeleton of the two fucoidan consists of α-(1 → 3)-linked L-Fucp residues, primarily sulfated at the C-2 and C-4 positions. The side chains include terminally linked α-L-Fucp and α-D-Galp residues, with (1 → 2)-, (1 → 6)-, and (1 → 2,6)-linked *β*-D-Galp residues mainly attached to the O-4 position of the main chain residues. In addition, studies have shown that SHAP-1 and SHAP-2 can exert anti HSV activity by preventing HSV-2 from adsorbing onto the host cell membrane in the early stage of infection. It can be concluded that the antiviral mechanism of fucoidan is to block the adsorption of HSV-2 virus particles on host cells (57). The aqueous alcohol extract of *Sargassum* showed antiviral activity against the highly prevalent human enterovirus Echovirus 9. Through qualitative determination of the type of secondary metabolites in the extract and evaluation of cytotoxicity, the quinones, proanthocyanidins, catechins, polar triterpenes, hydrolyzable tannins, etc. were found to strongly inhibit the replication of Echovirus 9 (58). Fucoidan exhibits a range of biological activities, including *in vitro* resistance to various RNA and DNA viruses, particularly to important human pathogens such as HSV-1, HSV-2, cytomegalovirus, dengue virus and HIV (59). Fucose is a compound with complex structure, which has antiviral activity due to its direct interaction with envelope virus (60). Dinesh et al.’s experiment showed that the fucoidan components have inhibitory activity against HIV-1 *in vitro* (61). Moulard et al. have proved that fucose exerts its antiviral effects by binding to HIV-1, thereby blocking the initial entry stage of the virus (62). These macromolecules may shield the positively charged amino acids in the viral envelope glycoprotein gp120 or strongly bind with specific sulfate groups, thereby inhibiting HIV-1 activity (62, 63). Akamatsu et al. isolated a novel fucosan polysaccharide named MC26 from the marine brown alga species *Sargassum*. MC26 demonstrated potent activity against the influenza virus while exhibiting low cytotoxicity (64). Mandal et al. extracted fucoidan sulfate from brown seaweed, and found that the fucoidan can effectively combat HSV-1 and HSV-2, while showing no cytotoxic effects in Vero cell cultures. Copolysaccharides mainly inhibit the formation of virus induced syncytium by suppressing virus-cell interactions, thereby proving their antiviral activity (65, 66).

A fucoidan (KW) extracted from the brown alga *Kjellmaniella crassifolia*, showed excellent activity against influenza IAV (67). KW demonstrated a broad-spectrum antiviral effect against IAV and a low tendency to induce viral drug resistance. It effectively inhibited IAV infection with low toxicity *in vitro* (68). KW effectively inactivated virus particles, prevented virus transmission after attachment, and inhibited the activity of virus neuraminidase binding, thereby blocking virus release (69). In terms of host regulation, KW effectively inhibited the activation of pathways including PKCα, NF-κB and EGFR, and inhibited viral endocytosis (70). Intranasal administration of KW to mice markedly enhanced the survival rate and lowered the viral titer following IAV infection (71).

In recent decades, *Sargassum fusiforme* polysaccharide (SFP) has attracted wide attention due to its antioxidant, immune regulation, anti-tumor, anti-aging and hypoglycemic effects (72–75). Some studies have found that SFPs with different molecular weights can combine with viruses and show good antiviral activity *in vitro*. After treatment with SFP, the expression of virus gene and protein decreased significantly, and the 9 kDa molecular weight SFP-3 showed the best antiviral effect (44, 76). Surprisingly, SFP in chickens showed effects in reducing immune suppression, inhibiting shedding, and minimizing organ damage caused by viral infections. To further improve the antiviral effect of SFP, there are studies using nanoparticle technology to prepare hydrophobic SFP into three kinds of nanomicelles (SFP-C12M, SFP-C14M, and SFP-C16M), which can enhance the antiviral effect of SFP. Compared with SFP, all three types of micelles showed better antiviral effect, with the SFP-C12M being the most stable and the SFP-C16M showing the best antiviral effect, which also exhibited activity during the virus replication stage. Subsequent giant unilamellar vesicle exposure experiment found that the antiviral activity of nano micelles may act on the phospholipid membrane of ALV-L virus, and offer a novel approach for developing antiviral drugs (77, 78).

### 3.3 Sulfated polysaccharide extracted from green algae

Ulvan is a significant water-soluble polysaccharide present in green seaweeds belonging to the Ulvales order, such as *Enteromorpha* and *Ulva lactuca*. The main components are xylose, rhamnose, sulfate, glucuronic acid and iduronic acid (79). Lahaye et al. showed that both natural and chemically modified formulations of *Ulva lactuca* contain many oligosaccharide repeating units, with the main repeating disaccharide unit being in the form of 3-sulfate composed of iduronic acid or glucuronic acid. This demonstrates that the structure of *Ulva lactuca* has great complexity and variability (80). Ulvan, accounts for 8–29% of the dry weight of green seaweed and is the most prevalent polysaccharide found in its cell walls. Studies conducted both *in vitro* and *in vivo* have demonstrated that ulvan possesses antibacterial, anticoagulant, immunomodulatory properties and antiviral (80–85). Studies have shown that *Ulva lactuca* can effectively suppress the measles virus (MeV) by limiting the formation of syncytia, and inhibit HSV virus by inhibiting DNA replication and transcription, and downregulating HSV protein synthesis. The edible blue-green algae *Nostoc flagelliforme* contains the acidic polymer known as *Nostoc flagelliforme* polysaccharide (NSF). Kanekiyo et al. discovered that NSF can effectively inhibit a number of enveloped viruses. Studies have confirmed that NSF can achieve anti-herpes effect by inhibiting the combination of virus and host cells, and *Nostoc flagelliforme* polysaccharide can be used as a candidate drug for anti-herpes (86).

The mosquito-borne flavivirus known as the Japanese encephalitis virus (JEV) kills many people in Southeast Asia each year and produces a significant number of encephalitis cases (87, 88). But effective drugs to inhibit JEV are still lacking, so the development of cheap and easily available antiviral drugs with low side effects is urgently needed (89). One of the experiments documented treatment impact on JEV infection with the aid of *U. lactuca* sulfated polysaccharide extract. Chiu et al. found sulfated polysaccharides in *Ulva lactuca* had antiviral and anti-inflammatory properties toward Vero cells infected by JEV and mixed primary glial cells upon treatment *in vitro* and inspected antiviral action under electron microscopy. Then, the antiviral effect of a sulfated polysaccharide extract of *Ulva lactuca* was then investigated in C3H/HeN mice infected with JEV (89). The results are also similar to other sulfated polysaccharides, which are more suitable as preventive agents rather than therapeutic agents and can also interfere with the binding of viruses to cells. However, examination of the mixture by virus binding assay combined with transmission electron microscopy revealed that the extracts could bind to JEV as a 110 nm sized complex, indicating that the *Ulva* sulfated polysaccharide extracts could adsorb JEV particles, thereby preventing the virus from entering cells. Unfortunately, this sulfate polysaccharide appears to be selective toward the Flaviviridae and does not bind to dengue viruses and West Nile viruses of the same genus. In the JEV model of murine infection, the antiviral effect of carrageenan was more remarkable. Encephalitis appeared 5 days after infection and all mice died shortly after indicating that carrageenan treatment not only delayed the onset time but also improved the survival rate of C3H/HeN mice (90). In the examination of host brain indicators, it was found that carrageenan was able to reduce the mRNA and protein expression levels of JEV, and significantly decreased the proinflammatory cytokines such as iNOS and TNF *α* (91–93). Sulfated polysaccharides have been shown to inhibit viral infections through multiple mechanisms, including blocking viral receptors and interfering with intracellular replication steps. These polysaccharides can prevent viral attachment by competitively binding to cell surface receptors or viral glycoproteins, thereby reducing viral entry. Additionally, they may disrupt key stages of viral replication, such as inhibiting viral RNA synthesis or interfering with viral protein processing, ultimately limiting viral propagation (94).

## 4 Anti-viral mechanism of marine polysaccharides

**Figure 3:**
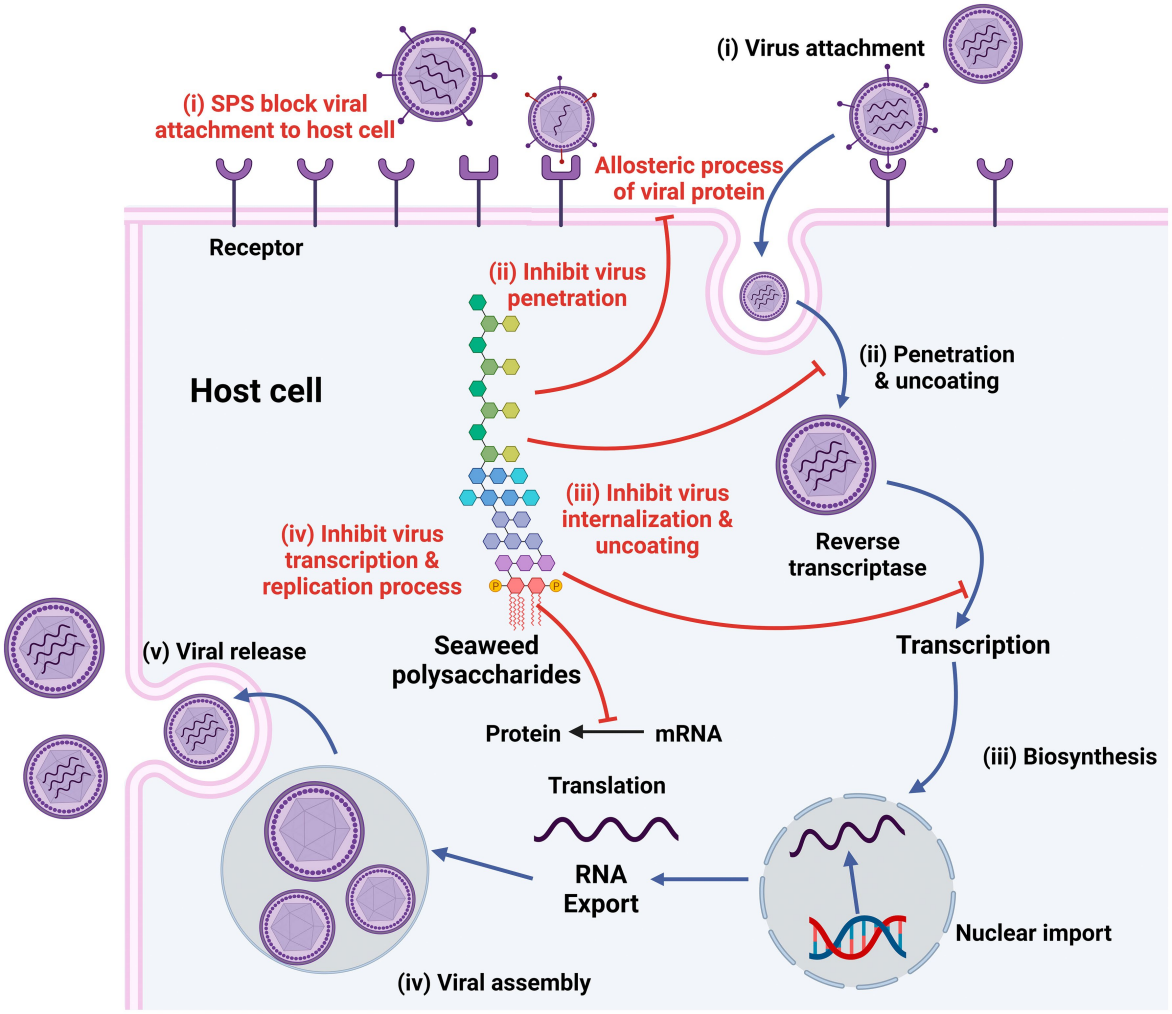
Stages in life cycle of virus (Black) and mechanism of antiviral actions of sulfated polysaccharides (SPs; Red). Created with BioRender.com.

Sulfated polysaccharides from algae have unique structures that exhibit antiviral properties. They operate at various stages of the viral life cycle either by directly inactivating virions before infection or by suppressing replication of the virus in host cells. Polysaccharide-rich algae thus play a huge role in antiviral drug discovery and development. The major steps in the viral life cycle—attachment, penetration, uncoating, biosynthesis, assembly, and release—vary from species to species (Figure 3) (95). Besides disrupting the life cycle of the virus and suppressing the replication of viruses, algal polysaccharides also stimulate the host’s antiviral immune response and accelerate virus clearance. Thus, algal polysaccharides can inhibit virus life cycles at different stages of a virus’s life cycle or can directly inactivate virus particles before virus infection. Typically, the mode of antiviral action is associated with specific structural characteristics of polysaccharides and particular viral serotypes (66).

### 4.1 Direct inactivation effects on virus

Due to the negative charge, sulfated polysaccharides can act directly on virus particle surfaces, thus inhibiting virus infectivity or killing the virus. Sulfated polysaccharides from marine sources can have the capacity to bind with spike glycoprotein, preventing SARS-CoV-2 from entering host cells (96). By targeting viral envelope glycoproteins, sulfated alginate polysaccharides of *Laminaria japonica* were shown to suppress HSV-1 at low concentrations by preventing the infection process and blocking the virus from detecting surface receptors on the host cell (97). Numerous studies further demonstrate that one of the modes of action of carrageenan is that it exerts a direct virus-killing effect on some enveloped viruses, which means that the virus loses its infecting ability, hence reducing virus reproduction. *λ*-carrageenan has the ability to bind strongly to herpes simplex virus, thereby inactivating HSV particles and inhibiting the replication of HSV. Furthermore, in low doses, the red algal polysaccharide carrageenan can directly inactivate HSV-2 (7, 98). The direct killing effect of the virus maybe because carrageenan exerts some sort of stable virus particle-carrageenan complex where the binding itself is irreversible. These sulfated polysaccharides occupy the location on the viral envelope where the virus must connect to the host cell, making it unable to carry out its following infection process (66). Namely, the polysaccharide of carrageenan can kill viruses directly and then prevent virus infection.

### 4.2 Inhibiting virus adsorption

**Figure 4:**
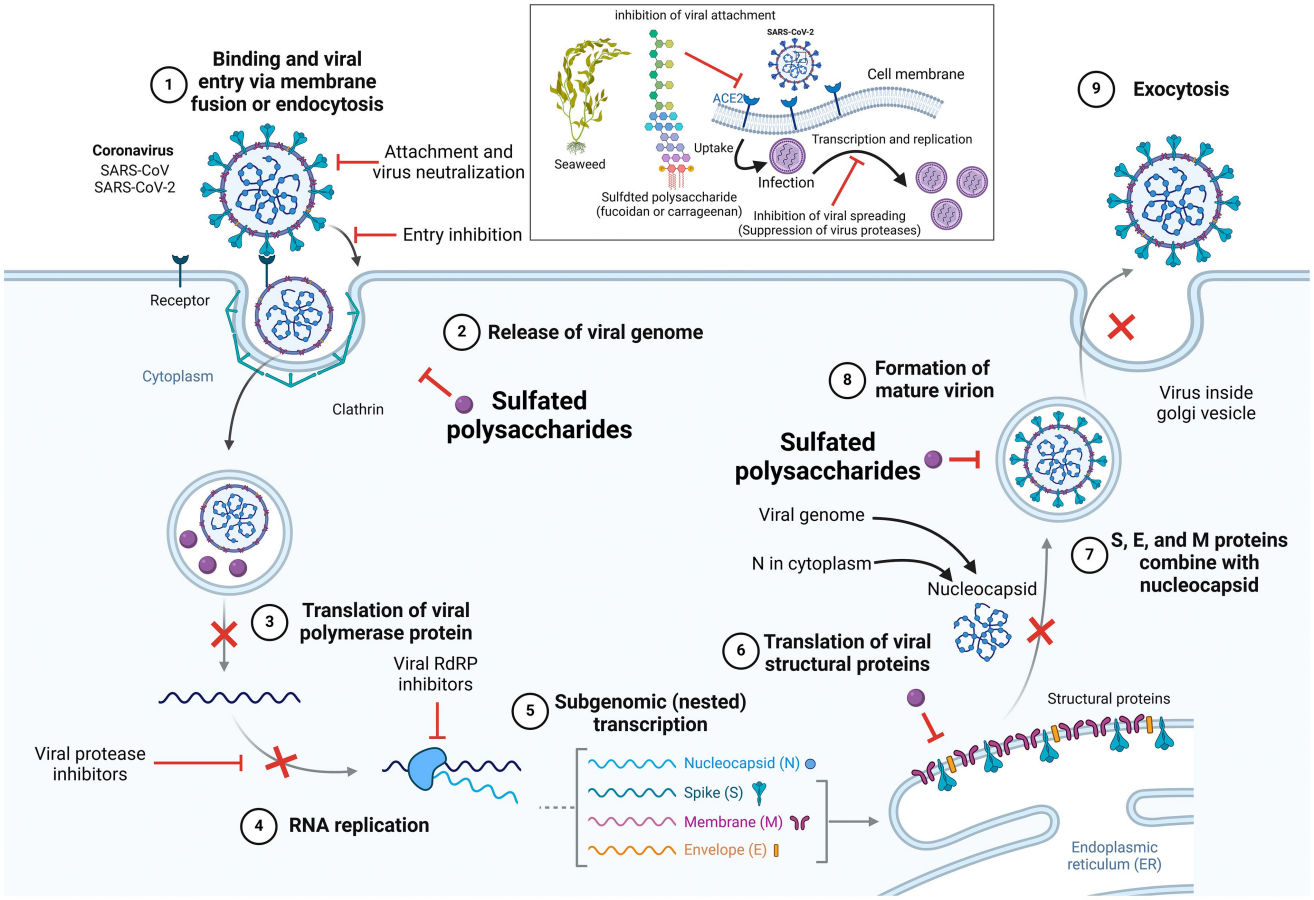
SARS-CoV-2 entry and replication cycle, with potential inhibition sites where seaweed polysaccharides may exert antiviral activity. Created with BioRender.com.

For infection to occur, viruses must adsorb and penetrate target cells. The first step in the viral invasion process is the interaction with the host cell surface through electrostatic forces. The unstable reversible binding is then changed into stable irreversible adsorption in order to carry out the subsequent steps. Natural and synthesized sulfated polysaccharides have high polyanion characteristics. They can make the adsorption of viruses or host cellular surface proteins hinder through electrostatic contact with their cells, consequently blocking the binding site of viruses and host cellular receptors (99). Figure 4 depicts the SARS-CoV-2 entrance and replication cycle, along with putative inhibitory sites for marine-derived metabolites. The virus connects to the ACE2 receptor, then penetrates the cell and releases its RNA. The RNA generates viral proteins, which form new virus particles. These particles are processed in the endoplasmic reticulum-Golgi pathway and then released to infect new cells, particularly those that express ACE2 receptors in organs such as the heart, lungs, intestines and kidneys (100–102). Marine-derived natural compounds, such as seaweed polysaccharides, exhibit antiviral activity through two main mechanisms: inhibition of viral entry and replication. The S glycoprotein is a primary antigen on the virus surface, mediating necessary attachment and membrane fusion for cellular entry. Therefore, antiviral compounds can block this process by inhibiting the attachment of S protein to ACE2 receptors in a dose-dependent manner. Additionally, viral proteases like 3CLpro and PLpro, as well as RdRp, facilitate viral replication and transcription. By targeting key viral proteins such as N and M, these compounds can further suppress viral replication and block the spread of infection (103, 104). Furthermore, increased furin expression enhances MERS-CoV pseudovirion infection, while furin siRNA silencing reduces furin expression and subsequently viral entry. Fucoidans inhibit coronaviruses by targeting both the viral spike protein and the host cell furin, thereby interfering with viral infection (105, 106).

Carrageenan is capable of masking the positive charge on the host cell surface by its negative sulfate group charge, thus interfering with the process of virus adsorption. Mazumder et al. isolated high molecular weight sulfated galactose from red algae, which can prevent the initial attachment of viruses to host cells and demonstrates activity against herpes HSV-1 and HSV-2 (107). Then, Kalucci et al. verified that *λ*-carrageenan might obstruct the virus’s ability to adhere to host cell surfaces (90, 108). Alginate glucan produced from brown algae has been shown to have strong antiviral properties against a variety of viruses, including the human HSV and HIV. Fucoidan acts by inhibition of viral particles binding to host cells, thus activating its antiviral activity (109). Moreover, sulfated polymannurate (SPMG) is a new sulfated marine polysaccharide derived from brown algae that can prevent the penetration and adsorption of HIV-1 by competitively sharing a gp120 common binding site with sCD4 or masking its gp120 docking site on the T lymphocyte surface with sCD4 (110). Sulfated fucoidan polysaccharide cotyledons obtained from nodular seaweed have been reported to give a very significant inhibition against the early stages of HBV, HCV and HIV-1 infection but show no effect on the late stages of the same virus infection (19). Alginate has a direct interaction with the envelope glycoprotein on the surface of dengue virus DEN2 (66). Furthermore, by blocking the virus’s contact with host cells, the acidic polysaccharide Nostoflan, which is extracted from the edible blue-green algae *Nostoc flagellate*, also showed a good suppression of HSV-1 production (86). In summary, the adsorption step of virus infection could be interfered with by marine polysaccharides originated from various sources to prevent virus infection.

### 4.3 Inhibition of viral transcription and replication

After internalizing into host cells, algal polysaccharides, particularly low molecular weight algal oligosaccharides, can impede transcription and replication in addition to blocking the viral invasion process. The sulfated polysaccharide chains have some identical binding sites with some RNA template primers-related enzymes, leading to their competitive inhibition. However, by acting on similar targets inside intracellular targets, sulfated polysaccharides directly affect replication-related enzymes and prevent transcription and replication. Thus, Talarico et al. demonstrated that by interfering with possible host cell targets, *ι*-carrageenan may prevent DENV multiplication in mosquito cells (111). Furthermore, carrageenan oligosaccharides with tiny molecular weights can enter host cells to prevent transcription and viral multiplication. Low molecular weight *κ*-carrageenan oligosaccharides have been found by Wang et al. to successfully suppress influenza A H1N1 virus multiplication *in vitro* and *in vivo* (49). Additionally, certain marine polysaccharides derived from brown algae prevent viruses from replicating in host cells. At a dose of 0.5–1.0 mg/mL, Queiroz et al. discovered that the fucans isolated from *Fusarium vesiculatum* demonstrated a notable inhibitory impact on HIV reverse transcriptase *in vitro*. Furthermore, alginate derivative 911 can not only suppress HIV-1 reverse transcriptase activity but also inhibit virus adsorption. Conclusively, marine polysaccharides could interfere with viral replicase or other potential targets in the host cell to inhibit viral transcription and replication (112–114).

### 4.4 Improve host antiviral immune response

Proceeding from the pathogenesis of the disease, pathogenic treatment for viral infection should focus on the virus directly or on the process of its adsorption and penetration into cells, as well as on innate immunity activation, antioxidant defense system strengthening, immune cytokine production, and indirect antiviral effects (115). Many pharmacological experiments have demonstrated that sulfated polysaccharide is a potent immune regulator in an attempt to maintain the integrity of body balance via a series of immunomodulatory regulation activities against natural killer cells (NK cells), macrophages, T/B lymphocytes, and other immune cells through the promotion of cytokine release and antibody production, which may indirectly activate the complement system, inhibit virus replication, and accelerate the virus clearance process (26, 111). In macrophages, they promote immune responses by upregulating the production of nitric oxide, interleukin-6, prostaglandin E2, interleukin-1β, and tumor necrosis factor-*α*. Additionally, they enhance the expression of key enzymes such as inducible nitric oxide synthase and cyclooxygenase-2, further amplifying the inflammatory response and immune defense mechanisms. The study’s findings demonstrated that giving mice carrageenan for 8 h causes them to produce type I interferon, as well as significantly enhances the NK cell activities and lymphocyte proliferation rate. In addition, the oligosaccharide of carrageenan may stimulate macrophages and NK cells activity, improving IL-2 and TNF-α (116, 117). These findings suggest that the antiviral effect of carrageenan is closely linked to its ability to strengthen the host immune system, indirectly inhibiting viral replication and facilitating virus clearance.

## 5 Conclusion

Marine polysaccharides, particularly those derived from red, brown, and green algae, have demonstrated significant antiviral properties. These sulfated polysaccharides exhibit broad-spectrum antiviral activities, including direct inactivation of viruses, inhibition of viral adsorption, suppression of viral transcription and replication, and enhancement of the host’s immune response. Notably, they possess low toxicity, minimal drug resistance, and excellent biocompatibility, making them promising candidates for the development of antiviral medications. For instance, carrageenan from red algae and fucoidan from brown algae have been proven effective against viruses such as influenza, HIV, and herpes simplex virus. Similarly, sulfated polysaccharides from green algae, like those found in *Ulva* species, exhibit antiviral properties against diseases such as the Japanese encephalitis virus. These polysaccharides function by inhibiting the attachment of viruses to host cells or by interfering with various stages of the viral life cycle. Furthermore, marine polysaccharides can boost the host’s immune responses and promote viral clearance.

While these findings underscore the antiviral potential of marine polysaccharides, most studies have been conducted *in vitro* or in animal models. Further clinical trials are necessary to validate their effectiveness and safety for therapeutic use. In addition, the structural complexity of marine polysaccharides poses difficulties in the standardization of extracts and formulations, which makes it challenging to establish consistent therapeutic applications. Although various antiviral mechanisms have been proposed, a comprehensive understanding of the precise molecular interactions between polysaccharides and viral components is still lacking. In addition, the natural origin of these compounds poses regulatory challenges, as they may not meet current standards required for drug approval. By addressing these challenges, marine polysaccharides could be a valuable addition to antiviral therapies, providing new solutions for the treatment of various viral infections.
